# SmileyLlama: modifying large language models for directed chemical space exploration

**DOI:** 10.1038/s43588-026-00986-y

**Published:** 2026-05-11

**Authors:** Joseph M. Cavanagh, Kunyang Sun, Andrew Gritsevskiy, Dorian Bagni, Yingze Wang, Thomas D. Bannister, Teresa Head-Gordon

**Affiliations:** 1 Kenneth S. Pitzer Theory Center and Department of Chemistry, University of California, Berkeley, Berkeley, CA USA; 2 Promontory Labs, San Francisco, CA USA; 3 Department of Molecular Medicine, The Herbert Wertheim UF Scripps Institute for Biomedical Innovation and Technology, Jupiter, FL USA; 4 Departments of Bioengineering and Chemical and Biomolecular Engineering, University of California, Berkeley, Berkeley, CA USA; 5 Chemical Sciences Division, Lawrence Berkeley National Laboratory, Berkeley, CA USA

**Keywords:** Method development, Computational models

## Abstract

Here we show that large language models (LLMs) can be transformed via supervised fine-tuning of engineered prompts into SmileyLlama for exploring the chemical space of drug molecules. We benchmark SmileyLlama against pretrained LLMs and chemical language models trained from scratch for generating valid and novel drug-like molecules, and use direct preference optimization to both improve SmileyLlama’s adherence to a prompt and as part of the iMiner reinforcement learning framework to predict molecules with optimized three-dimensional conformations and high binding affinity to drug targets. By training an LLM to speak directly as a chemical language model, while retaining most of its natural language capabilities, we show that SmileyLlama can reliably generate molecules with user-specified properties rather than acting only as a chatbot with knowledge of chemistry or as a virtual assistant. While SmileyLlama is geared toward drug discovery, the supervised fine-tuning/direct preference optimization/LLM framework can be extended to other chemical, biological and materials applications.

## Abstract (web summary)

A general-purpose large language model can be trained to create a specialized model, SmileyLlama, that can generate drug-like molecules with user-specified properties that are relevant to medicinal chemistry through a process that might be extended to other scientific domains.

## Main

Chemical language models (CLMs)^1^ trained on string representations such as Simplified Molecular-Input Line-Entry System (SMILES)^2^ and SELF-referencIng Embedded Strings (SELFIES)^3^ have emerged as a useful tool for de novo generation of molecules, best exemplified by molecules relevant to pharmaceutical applications and drug discovery^4–6^. Nearly all CLMs for molecular generation have been trained from scratch on large quantities of data such as ChEMBL^7^ and ZINC^8^ and using different model architectures, including variational autoencoders^9^, recurrent neural networks^10^, generative pretrained transformers (GPTs)^11^ and structured state space sequence (S4) models^12^. In addition, CLMs have achieved advances in chemical generation tasks through further downstream optimization of molecules with additional training or different model frameworks^13,14^.

Language models are statistical models of probability distributions of units of language and can be adapted to generate meaningful text by sampling from these distributions. The most recent advancement of language models have resulted from the training of scaled-up transformers^15^ on massive amounts of data, resulting in the creation of large language models (LLMs) such as the proprietary GPT-4^16^ and the open-weight Llama^17^. Recently scientific groups have accessed frontier LLMs for the purpose of assisting research in the form of virtual lab members, to translate between natural and chemical languages^18^, or even performing research autonomously^19–21^. Beyond chemical dictionary lookups or lab aides, LLMs have been used to perform mutation and crossover for an evolutionary algorithm to explore chemical space^22^ or to modify SMILES strings to change the properties of the molecules that they represent^23,24^. Others have taken inspiration from LLMs to design CLMs, such as the ability to respond to prompts through a transformer-based architecture^25^. However, to our knowledge, no CLM derived from a pretrained general-purpose LLM has reached the performance exhibited by modern CLMs that are trained from scratch with chemical data.

Here, we demonstrate that an open-weight LLM, Meta-Llama-3.1-8B-Instruct (‘Llama’ here on out)^17^, can be converted into a model for generative tasks in drug discovery. The fact that Llama is open-weight offers several benefits including allowing training and sharing adapters, to perform inference without needing to store potentially valuable data on a remote server, to have control over the hyperparameters and algorithms used for fine-tuning, and to perform interpretability analyses on the model weights. Using supervised fine-tuning (SFT) and direct preference optimization (DPO)^26^ of the pretrained Llama with SMILES strings derived from ChEMBL, SmileyLlama generates drug-like molecules with desirable properties specified in a user-defined prompt with relevance to medicinal chemistry, which we show can match or exceed the performance of modern CLMs. We further demonstrate that SmileyLlama greatly improves the reinforcement learning component of iMiner algorithm^14^ to more efficiently explore chemical space to create molecules optimized for three-dimensional (3D) binding to target proteins, illustrated with the SARS-Cov-2 (SARS2) main protease (MPro)^27^. While our dataset and subsequent analyses are created with drug discovery as a downstream application, this general procedure can be extended to other chemical applications such as chemical synthesis planning^28^ or transition metal complex discovery^29^.

## Results

### SFT and DPO of Llama

To steer the outputs of the pretrained Llama model^17^ for drug molecule generation, we first use SFT, in which the weights of Llama are further optimized on SMILES strings of approximately 2 million molecules from the ChEMBL Dataset (v33)^7^ to create SmileyLlama. For each molecule in our dataset, we picked a number of molecular properties of pharmaceutical interest to calculate using RDKit^30^ and that are relevant for medicinal chemistry. In addition, drug molecules must also have suitable characteristics related to relevant biological phenomena such as obeying the rule-of-five^31^, or topological polar surface area (TPSA) ranges that are associated with oral bioavailability or the ability to cross the placenta or the blood–brain barrier^32^. If a drug need not meet these criteria, then a user interfacing with SmileyLlama should also be able to adjust the range criterion or eliminate it. Further specifics of these properties and the ranges we choose to specify during training of SmileyLlama can be found in the ‘Details of properties for fine-tuning’ section in the Methods.

After calculating and picking these properties for each SMILES string, we construct a prompt containing values of these properties, with the ‘correct’ completion being the SMILES string that these properties were calculated from. To illustrate, we used a prompt with a system instruction of ‘You love and excel at generating SMILES strings of drug-like molecules’ and a user instruction of the form ‘Output a SMILES string for a drug like molecule with the following properties:’ if properties are specified, or ‘Output a SMILES string for a drug like molecule:’ if no properties are specified. We chose to create prompts that assign SmileyLlama the role of an artificial intelligence that excels at producing SMILES strings, given the effectiveness of role prompting^33^; we also chose this prompt format owing to its balance between motivation and brevity. Each property has a 50% chance of being calculated and specified in the prompt so that the trained model learns to operate equally well during inference, whether or not any properties are specified. We structure the prompts used for SFT so that during inference users avoid having to downselect the vast majority of generated molecules for having the correct characteristics—instead, users can simply prompt SmileyLlama to provide molecules with the characteristics they desire. See ‘Prompt formats and examples’ and ‘Additional training details’ sections in the Methods, as well as Supplementary Algorithm 1 and Supplementary Fig. 1 for further elaboration of SFT training.

We also use DPO^26^, which also updates the weights of Llama to reinforce our model’s ability to robustly generate molecules for more specific task-oriented goals such as property specification. Algorithmically, we prompt our SFT model to generate molecules with a given property, sample several SMILES strings and use RDKit^30^ to assess whether they have properties in line with what the prompt requested. We then pair molecules that correctly follow the prompt with those that do not, labeling them as winners and losers, respectively, and use a single epoch of DPO to improve the model’s performance. See Supplementary Algorithm 2 for pseudocode of this scoring and pairing procedure.

### Benchmarking SmileyLlama against other LLMs and CLMs

To test the generative ability of SmileyLlama compared with other existing CLMs, we used the GuacaMol suite^34^ to benchmark the validity, uniqueness and novelty of the molecules as shown in Table 1. In addition, Kullback–Leibler (KL) divergence and Fréchet ChemNet distance (FCD)^35^ based on the GuacaMol definition (FCD_Guac_) are used to analyze the distributional shifts from the ChEMBL training data for drug-like molecules^34^. More detail is found in the ‘GuacaMol benchmark definitions’ section in the Methods.

**Table 1:** GuacaMol benchmarks comparing SmileyLlama with LLMs and with common CLM architectures trained on ChEMBL

Benchmark	Validity	Uniqueness	Novelty	KL div	FCD_Guac_
GraphMCTS^69^	1.000	1.000	0.994	0.522	0.015
VGAE-MCTS^69^	1.000	1.000	1.000	0.659	0.009
AAE^69^	0.822	1.000	0.998	0.886	0.529
LSTM^12^	0.983	0.999	0.848	0.993	0.901
GPT^12^	0.915	1.000	0.978	0.977	0.826
S4^12^	0.971	0.997	0.961	0.994	0.853
Llama zero-shot	0.688	0.457	0.635	0.736	0.002
Llama twenty-shot	0.465	0.999	0.949	0.913	0.079
SmileyLlama	0.958	1.000	0.987	0.967	0.686

The model benchmarks include valid chemical molecules, uniqueness and novelty with respect to the training set, and distribution similarity evaluated using KL divergence and Frechet ChemNet distance based on the GaucaMol definition FCD_Guac_ = exp(−0.2 × FCD). Additional information is available in the ‘GuacaMol benchmark definitions’ section in the Methods.

We first analyze the ability of Llama to produce molecules, relying only on its pretrained knowledge (zero-shot), or by providing it with one or more examples from the ChEMBL database in the formulated prompt (Table 1 and Supplementary Table 1). We find that, without SFT or examples provided in the prompt, the LLM is unable to produce a high percentage of valid SMILES strings compared with other state-of-the-art CLMs and generally performs poorly, even with variations in hyperparameters such as temperature (*T*). Interestingly, validity is lower when 20 examples are provided in the prompt (twenty-shot) than it is when no examples are in the prompt (zero-shot). We speculate that Llama zero-shot has had some exposure to the SMILES syntax to be able to generate valid strings, but it has no intrinsic ability to generalize, repeating the memorized SMILES and resulting in low uniqueness. When several examples are given in the prompt, this biases Llama away from the known SMILES strings it can produce, but the there are few enough examples provided that its grasp on the allowed mutable structure of SMILES strings is poor and thus less valid. However, because these prompts are so diverse, Llama’s 20-shot uniqueness is very high.

In Table 1, it can be seen that SFT substantially improves SmileyLlama’s ability to generate drug-like molecules. In addition, we experiment with the format of the SmileyLlama prompt, performing SFT on Llama with a less anthropomorphic user prompt and a blank template as an ablation study, showing that changing this prompt format does not substantially affect the GuacaMol benchmarks (Supplementary Table 1). To show the generality of the LLM-SFT approach, we also fine-tune Llama-3.2-3B, Llama-3.2-1B and Qwen-2.5-7B^36^ using the same SFT workflow (including identical hyperparameters) that we developed for SmileyLlama. Supplementary Table 1 finds that the GuacaMol benchmark results did not change substantially between SmileyLlama and SmileyQwen2.5-7B. We also find, through inspection of SmileyLlama-1B and SmileyLlama-3B, that validity increases with parameter count, while novelty, uniqueness, and the match between the training distribution and the distribution of generated molecules remain largely unchanged.

**Fig. 1:**
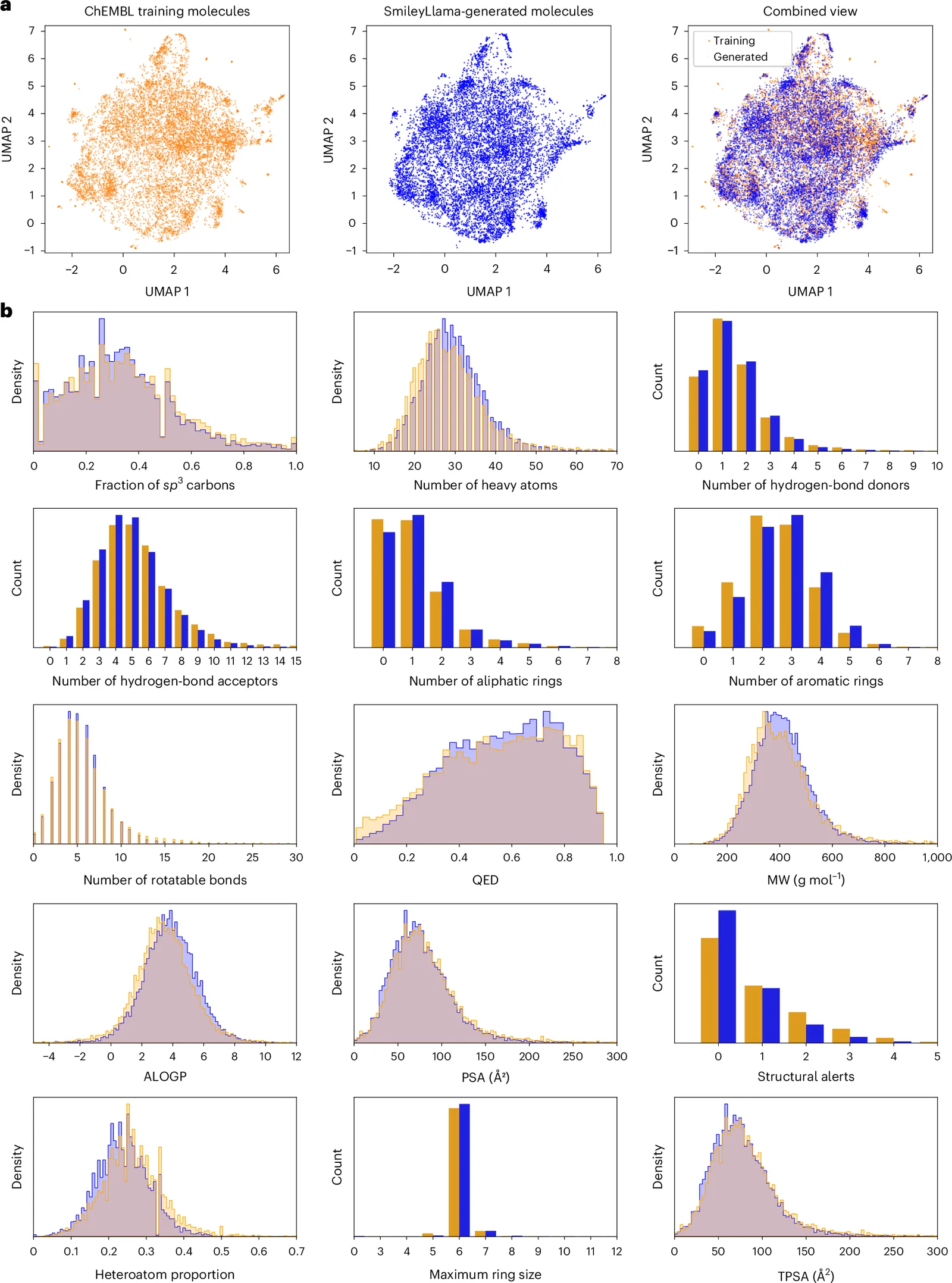
Distribution comparisons for different properties of the generated molecules from SmileyLlama (blue) with molecules from the training dataset from ChEMBL (gold). **a**, UMAP visualization of a random selection of 10,000 ChEMBL molecules and 10,000 SmileyLlama-generated molecules, using 15 neighbors and a minimum distance of 0.1; these are normal values in chemical space visualization^70^. **b**, The molecular properties considered are fraction of *sp*^3^-hybridized carbons and heteroatoms, number of heavy atoms, number of H-bond donors and acceptors, number of aliphatic and aromatic rings and the maximum ring size, number of rotatable bonds, QED value, MW, approximate log partition coefficient between octanol and water (ALOGP), polarizable surface area (PSA) and TPSA, and the number of structural alerts. All benchmarks were at a temperature *T* = 1.0 and a maximum of 256 new tokens. Source data

Figure 1 shows that SmileyLlama generates very good agreement with ChEMBL quantities across a diverse property set. The Uniform Manifold Approximation and Projection (UMAP) visualization in Fig. 1a, a popular visualization tool used in drug discovery, finds that SmileyLlama generates molecules in every well-represented region of the chemical space of ChEMBL. We also consider the distribution of molecular properties of interest to medicinal chemistry in Fig. 1b, where the KL-divergence values indicate that all properties are in strong agreement between SmileyLlama-generated molecules and ChEMBL molecules, and are comparable to those of other models, as reflected by low KL divergence in GuacaMol (Table 1 and Supplementary Figs. 3 and 4). Furthermore, small percentages of undesirable molecular scaffolds are present in the ChEMBL training data itself^14^, but Supplementary Table 2 shows that SmileyLlama and most robust CLMs do not oversample these unviable chemical structures. Finally, while training was conducted at *T* = 1.0, exploration of temperature used at inference on the GuacaMol benchmark (Supplementary Fig. 2) suggests that this temperature is adequate for all tests described in the ‘Results’.

### Property specification using SmileyLlama under SFT

In Table 2, we show the average percentage of valid, distinct SMILES strings generated for a complete panel of of molecular property tasks with SFT. This benchmark is distinct from other conditional molecule generation benchmarks^18^ in that we are testing SmileyLlama’s ability to robustly generate molecules with properties in value ranges rather than a specific value. This is of interest to medicinal chemists where numerical ranges of Lipinski violations or hydrogen bond donors and acceptors (and others) are used during chemical exploration. In addition, LLMs tend to struggle with numbers that have many degrees of precision and must be split into several tokens^37^. Hence, we did not represent this category in the prompt during training.

**Table 2:** Percentage of valid, distinct generated molecules over a panel of tasks using SmileyLlama

Present in trained prompt	SFT		DPO		Prompt ablation	
	*T* = 0.7	*T* = 1.0	*T* = 0.7	*T* = 1.0	*T* = 0.7	*T* = 1.0
≤*k* H-bond donors	96.6%	94.4%	99.2%	98.1 %	93.8%	91.8%
≤*k* H-bond acceptors	96.3%	90.7%	98.3%	98.4 %	76.6%	65.4%
≤*k* MW	90.1%	84.3%	97.8%	98.0 %	62.4%	58.8%
≤*k* ClogP	89.2%	85.4%	96.8%	97.7 %	67.9%	64.9%
Rotatable bonds in range	90.0%	86.7%	94.5%	94.0 %	62.2%	58.5%
Fraction *sp*^3^ in range	87.6%	85.0%	96.3%	95.9 %	30.7%	31.8%
TPSA in range	95.9%	91.1%	98.8%	98.4 %	89.2%	83.1%
No bad SMARTS	92.5%	89.6%	94.9%	94.3 %	87.3%	85.6%
Absence of macrocycle	98.4%	95.3%	98.4%	97.3 %	97.1%	94.8%
Absence of warhead-related SMARTS	96.4%	94.2%	97.3%	95.4 %	94.6%	92.9%
Lipinski rule-of-five	89.0%	81.7%	98.7%	97.7 %	73.6%	64.0%
Presence of warhead-related SMARTS	58.3%	51.0%	74.9%	73.0 %	0.2%	0.4%
Presence of Enamine substructures	51.0%	51.5%	67.7%	70.1 %	2.7%	1.9%
Presence of macrocycle	38.8%	44.7%	58.0%	57.8 %	2.1%	2.1%

Absent in trained prompt	*T* = 0.7	*T* = 1.0	*T* = 0.7	*T* = 1.0	*T* = 0.7	*T* = 1.0
Rule-of-three	77.5%	62.0%	84.8%	94.5%	7.5%	5.5%
Exactly *k* H-bond donors	21.4%	19.7%	30.7%	30.4%	17.4%	16.8%
Exactly *k* H-bond acceptors	19.9%	14.4%	27.0%	31.9%	9.2%	8.8%

For each task, we generate 1,000 molecules from a prompt requesting some property, and score the result based on the proportion of molecules that are valid, distinct and satisfy the properties requested in the prompt. Finally, we collect some tasks into families (such as those with different range values) and average their scores to produce the results shown in the table below. All benchmarks were at a temperature *T* = 1.0 and a maximum of 128 new tokens to increase computational efficiency. We also note that running SFT for two epochs rather than one did not seem to greatly affect the performance on these benchmarks.

**Fig. 2:**
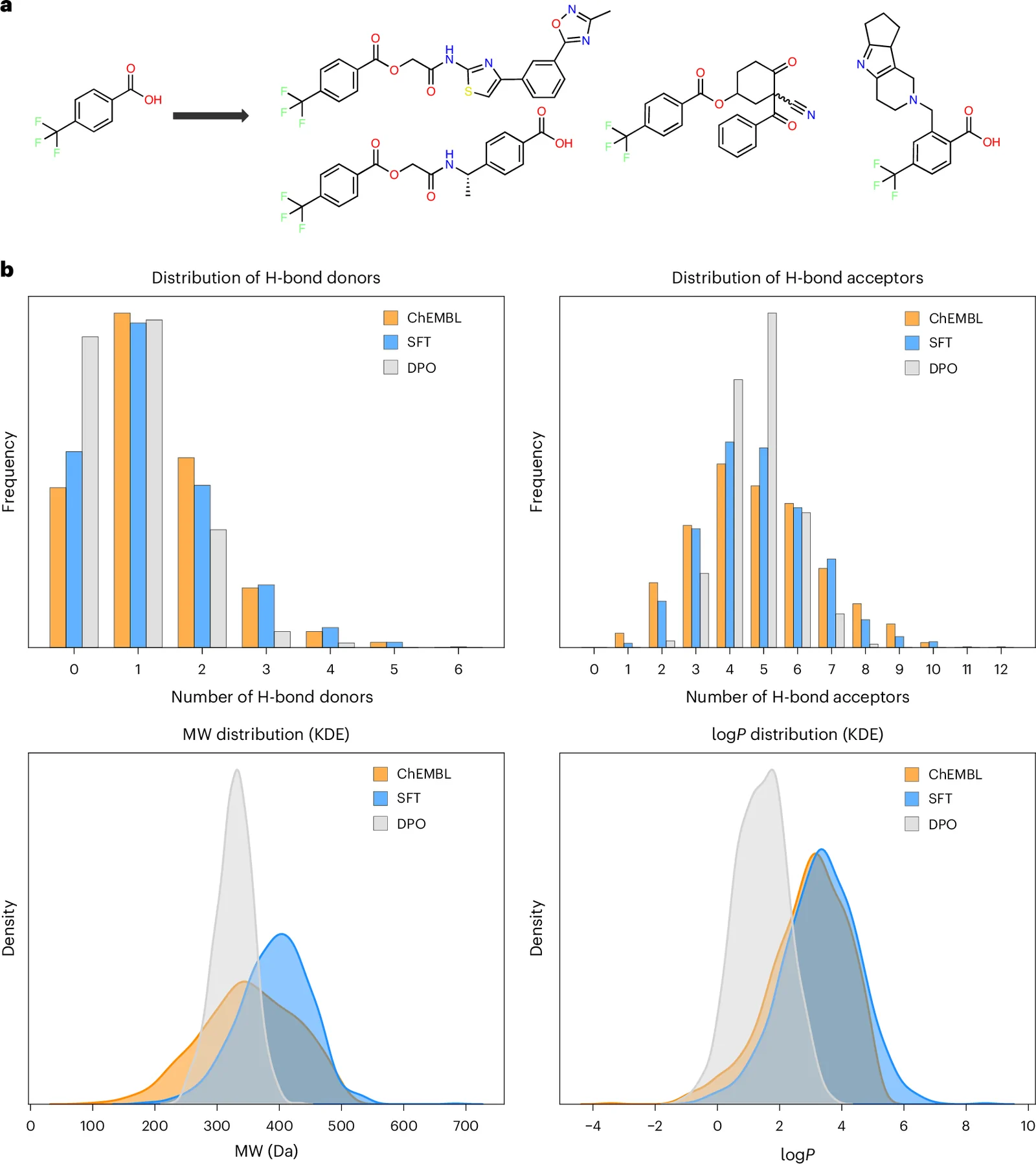
Conditional generation with SmileyLlama for fragment growth and before and after DPO compared with ChEMBL. **a**, Example molecules generated by growing from one of the Enamine substructures and to satisfy Lipinski’s rule-of-five using the prompt ‘Output a SMILES string for a drug like molecule with the following properties: a substructure of O=C(O)c1ccc(C(F)(F)F)cc1, < = 500 MW, < = 5 logP, < = 5 H-bond donors, < = 10 H-bond acceptors’. **b**, Distribution of four properties satisfying Lipinski’s rule-of-five comparing ChEMBL molecules (orange) with molecules generated by SmileyLlama (blue) with the prompt ‘Output a SMILES string for a drug like molecule with the following properties: < = 5 H-bond donors, < = 10 H-bond acceptors, < = 500 MW, < = 5 logP**’**, compared with 1,000 molecules generated by SmileyLlama with the same prompt after DPO (gray). MW and log*P* distributions were estimated using a Gaussian kernel density estimator (KDE). All results generated 1,000 molecules at a temperature *T* = 1.0 and a maximum of 128 new tokens. Source data

Overall, SmileyLlama model does very well on tasks on which it was trained through the engineered prompt, especially when contrasted with the model resulting from the ‘prompt ablation’ experiment in Table 2. We note that one has a choice to use SmileyLlama using lower temperatures at inference that can improve the SFT predictions further. Although all individual properties were present in the training data, some were underrepresented, such as the Lipinski rule-of-five, the presence of macrocycles, and certain categories of warhead-related SMARTS and Enamine substructures, resulting in more moderate performance for these categories. As expected, SmileyLlama does poorly on tasks involving exact numerical specifications. More encouragingly, SmileyLlama performs well on compound tasks such as generating molecules similar to existing leads, that is, ‘scaffold hopping’ R-group modification, and/or structure-based design to grow molecules from ligand fragments. Figure 2a is an example of SmileyLlama model’s ability to generate molecules from all 320 substructures in the Enamine database^38^ that follow the Lipinski rule-of-five^39^, which encompasses most of the molecular properties with ranges listed in Table 2.

We compare SmileyLlama with a model resulting from an ablation study on the efficacy of prompting. We study this by removing all indications of molecular properties from all of the prompts in the dataset used to train SmileyLlama; each molecule from ChEMBL is treated as a completion to the same prompt, namely the prompt used for SmileyLlama when no molecular properties are specified. When we run SFT with exactly the same hyperparameters as SmileyLlama, we find that the ablated model performs quite poorly in comparison to SmileyLlama on this benchmark, achieving 90+% performance on only three tasks. This becomes especially pronounced when the properties are rarely found in the data, such as the presence of a macrocycle or a warhead-related SMARTS pattern. The stark contrast in performance highlights the necessity of our prompt engineering scheme: we cannot rely solely on the knowledge of the foundation model when fine-tuning for chemical tasks.

### Property specification using SmileyLlama under DPO

While SmileyLlama typically performs well on tasks it was trained on using engineered prompts, and can still perform adequately when queried with prompts different from those it was trained on, it can be further optimized for specific tasks using DPO. DPO’s most popular application has been in improving the responses of LLM-derived chatbots, but it has also found use in improving the outputs of CLMs^40^ and avoiding the need to separately train a reward model^26^. Here, the relevance of DPO provides a way to further optimize the model by pairing desirable responses with undesirable responses. The model’s weights are then updated to be more likely to produce the ‘winner’ of the pairing and less likely to produce the ‘loser’ of the pairing. We generated our dataset by simply pairing up unsuccessful attempts at generating structures with successful attempts randomly for each task in Table 2.

SmileyLlama optimized with DPO substantially improved adherence to the prompt across nearly all tasks as seen in Table 2 and Fig. 2b. Note that, while DPO does cause the model to more robustly obey the rules in the prompt, it also shifts and narrows the property distribution compared to the training set and appears to be largely insensitive to temperature. SmileyLlama without DPO, on the other hand, occasionally does not obey the prompt but more faithfully reproduces the distribution of properties found in a filtered ChEMBL that satisfy Lipinski’s rules. In the context of drug discovery, SFT is primarily beneficial for early exploration of chemical space, whereas DPO is a type of constraint optimization that limits generated molecules to desired subclasses specified by the user.

### Binding affinity to protein active sites with SmileyLlama/iMiner

The tests performed in previous sections do not take advantage of the 3D structural information of a putative drug nor its shape and molecular compatibility with a target protein active site. Hence, we use SmileyLlama augmented with DPO to generate unique and valid ligands that undergo further optimization for binding to a specific protein target when embedded in the iMiner framework^14^. iMiner combined with SmileyLlama is designed to generate novel inhibitor molecules for target proteins by combining deep reinforcement learning^41,42^ with real-time 3D molecular docking using AutoDock Vina^43^, thereby simultaneously creating chemical novelty while constraining molecules for shape and molecular compatibility with target active sites. Further details of the iMiner reinforcement learning model have been published elsewhere^14^ and are briefly summarized in the ‘iMiner reinforcement learning with SmileyLlama’ section in the Methods. To validate the effectiveness of SmileyLlama in the iMiner context, we generate inhibitor molecules for MPro, an enzyme whose function is essential to the SARS2 lifecycle^44^. MPro has readily available experimental 3D structures^44,45^, which provide the information needed for structure-based ligand design.

**Fig. 3:**
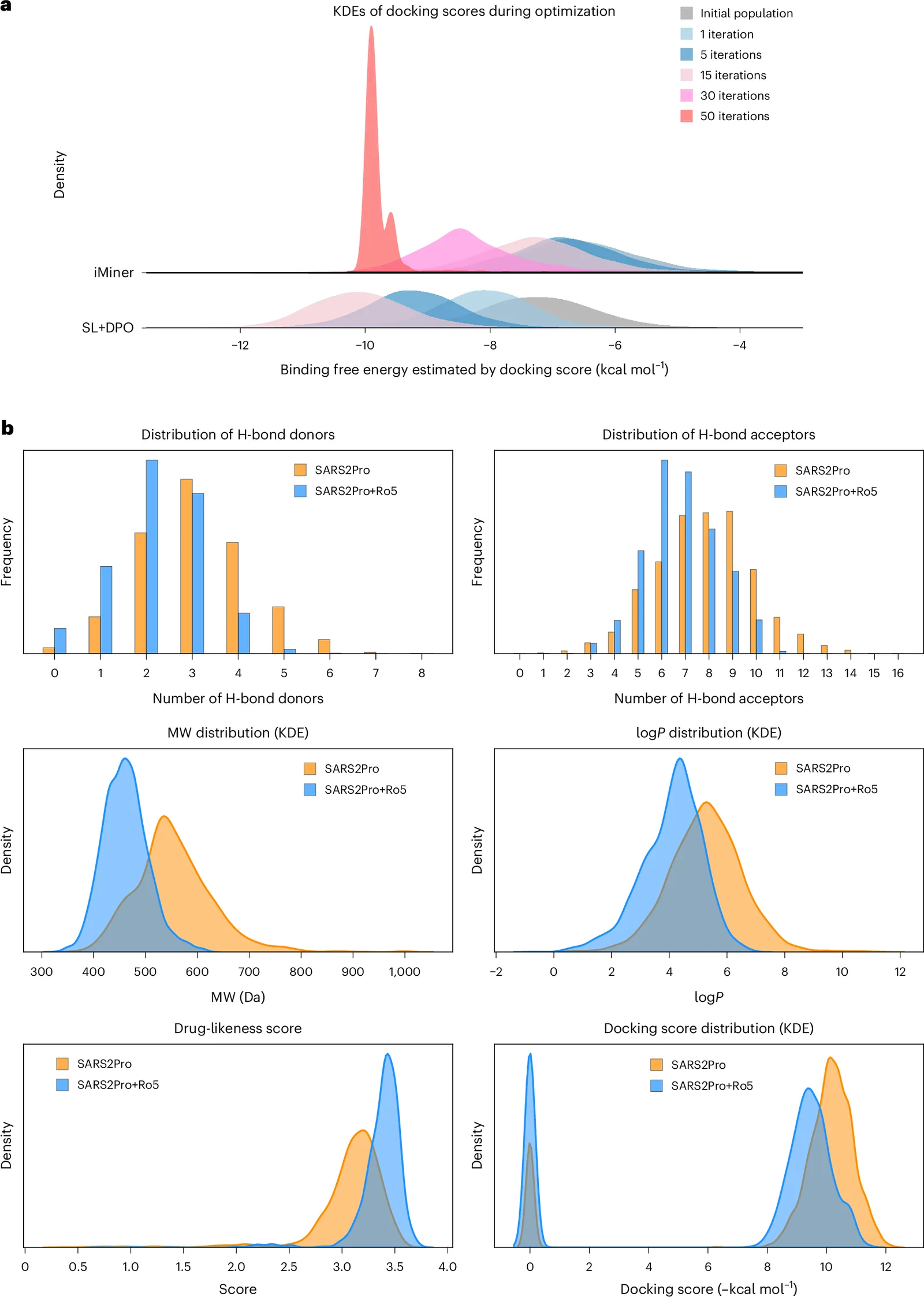
Comparison of the shift in docking score distributions for iMiner compared with SmileyLlama over optimization epochs as illustrated for SARS2 MPro. **a**, For iMiner, in later epochs, diversity crashes, which explains the sharpening peaks in later iterations. SmileyLlama with SL+DPO enforces diversity throughout the optimizations, which accounts for the broad peaks, and shows superior data efficiency relative to iMiner. **b**, We compare two different user prompts: Sars2Pro and Sars2Pro+Ro5. All results were generated with 2,000 valid SMILES at a temperature of *T* = 1.0 and a maximum of 128 new tokens. Source data

For the unconditional de novo generation case, SmileyLlama learns the user prompt ‘Output a SMILES string for a drug like molecule with the following properties: High SARS2PRO’, which pertains to minimizing the AutoDock Vina score while maximizing the drug likeliness score (*S*_DL_) of the original iMiner reward function^14^. Figure 3a compares the docking scores of the original iMiner algorithm against SmileyLlama as a function of epoch number and with number of generated molecules per iteration. We notice first an improved data efficiency compared to iMiner, in which SmileyLlama requires only ~25% of the epochs to reach a similar level of improved docking score. Furthermore, iMiner’s diversity crashes with more epochs, which explains the sharpening peaks in later iterations (Fig. 3) and quantified further in Supplementary Fig. 2 against the GuacaMol benchmarks. This simply reflects convergence in the docking score, that is, there are fewer novel molecules as docking score reaches the highest values. By contrast, SmileyLlama maintains more diversity while greatly improving the docking score to iMiner (Fig. 3a) with minor degradation in validity compared with iMiner (Supplementary Fig. 5).

Figure 3b shows the property distributions of the final optimized set of novel molecules from SmileyLlama from the above prompt. While the property distributions are satisfactory for the number of hydrogen bond donors and acceptors, the molecular weight (MW) and log*P* results are not conforming to drug-like values. This indicates some inadequacy of the iMiner reward function, such that the CLM would require a reweighting and/or new terms in the loss/reward function, other hyperparameter tuning and/or expensive retraining. However, a unique advantage of SmileyLlama is that the distribution of generated molecules’ properties can be shifted using nothing more than prompt engineering, with no retraining required. Figure 3b shows that combining prompts such as ‘Output a SMILES string for a drug like molecule with the following properties: High SARS2PRO, < = 5 H-bond donors, < = 10 H-bond acceptors, < = 500 molecular weight, < = 5 logP (High SARS2Pro+Ro5)’ improves properties such as MW and log*P* and drug-likeness scores substantially with some expected loss in high docking scores because smaller molecules make fewer intermolecular interactions.

**Fig. 4:**
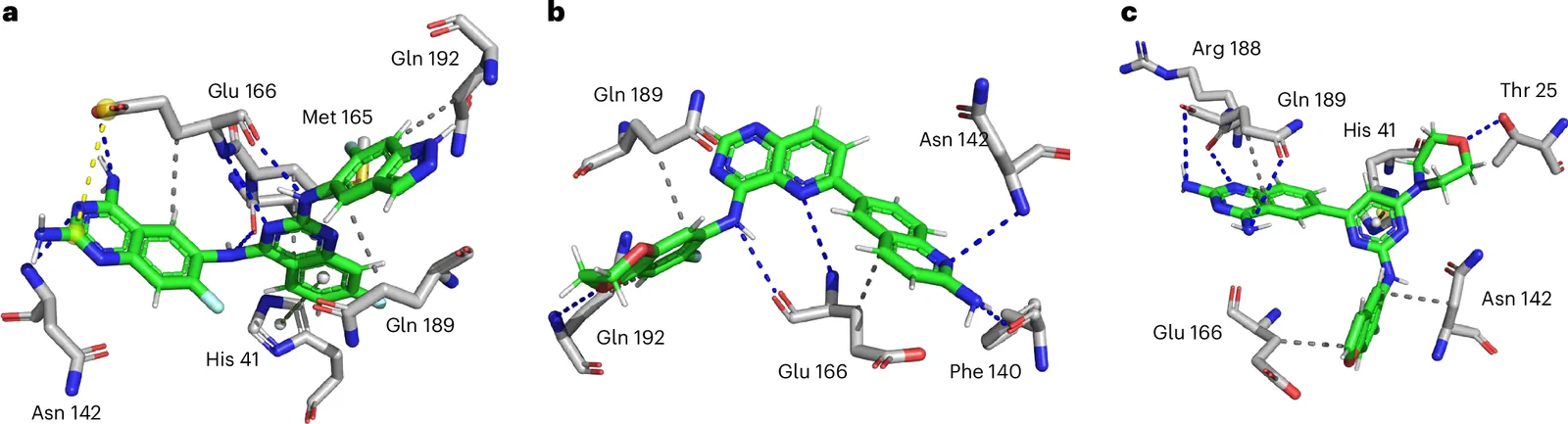
SmileyLlama de novo generated molecules in the active site of SARS2 MPro. **a**–**c**, Surface rendering of sample SmileyLlama-generated molecules in the SARS2 MPro canonical binding pocket. Generated by SmileyLlama after optimization with the SARS2PRO prompt (**a**) and two independent samples generated with the SARS2Pro+Ro5 prompt (**b** and **c**). The ‘molecule_examples.csv’ file in the Supplementary Data provides their SMILES string and docking scores, and Supplementary Fig. 6 shows the docking pose for some of the highest-scoring ligands. Blue is nitrogen, red is oxygen, green indicates ligand carbons and light gray indicates residue carbons.

Figure 4 and Supplementary Fig. 6 provides a set of novel molecules from SmileyLlama docked in the MPro active site with the two engineered prompts ‘High SARS2Pro’ and ‘High SARS2Pro+Ro5’. Two of the higher-scoring molecules resemble the variations of the perampanel drug with the trefoil structure, which are tested inhibitors optimized by the Jorgensen group^46^. However, unlike the molecules from their study that consistently retained the central pyridinone ring^46^, SmileyLlama molecules have replaced the trefoil hub with the pyrimidine functional group (Fig. 4c). Higher docking scores are found for quite different drug scaffolds (Fig. 4a,b), but in all cases there is no notable homology match found in the Therapeutic Target Database^47^. This would indicate that the generative capabilities of SmileyLlama are robust and outside of the pretrained Llama model. Finally, the proposed drugs are synthetically accessible^48^, as indicated by an average synthetic accessibility (SA) score of approximately 3. Precise details can be found in the ‘example_molecules.csv’ file in the Supplementary Data.

### SmileyLlama outside of chemical language modeling

While SFT and DPO alters Llama in the creation of SmileyLlama, we find that SmileyLlama can still converse in English if it is prompted to do so, and some sample conversations are included in the ‘SmileyLlama outside of chemical language modeling’ section in the Methods. As a more quantitative measure of its residual capabilities, we evaluate SmileyLlama’s performance using the Language Model Evaluation Harness on the MMLU, GPQA, Math-Hard, and MMLU-Pro benchmarks^49–53^. Supplementary Table 3 and Supplementary Fig. 7 show that SmileyLlama generally performs worse on moral scenarios and, interestingly, also performs worse on chemistry-related subjects than Llama. This is in part probably due to the tendency for SmileyLlama to complete prompts relating to chemistry with a SMILES string. In addition, accuracy errors in the MMLU tests have also been noted recently^54^, and thus SmileyLlama’s degraded performance in chemistry may be partly an artifact of poorly designed evaluation benchmarks. Overall, this result is somewhat encouraging, because it implies the possibility that LLM-derived CLMs can inherit and take advantage of the natural language processing ability of their foundation model. SmileyLlama already does this—we can steer the properties of the molecules it generates and the chemical space it explores using natural language prompts while still retaining some ability to process nonchemical natural language. However, more work is required to develop SmileyLlama as an additional capability of an LLM, which may be achievable with larger foundation models

## Discussion

Our study clarifies a few crucial points for CLMs derived from LLMs going forward. First, it is not necessary to pretrain a specialized model on chemistry-specific text to generate molecules from a text description; a much less resource-intensive SFT training run on prompt-following using a dataset of a few million molecules with a commodity LLM is sufficient to achieve this. Second, DPO provides another resource-efficient way of optimizing the model to produce molecules that score well on a targeted objective without needing in-context examples, instead relying on the generative nature of the model itself for good and bad examples. A corollary to this is the finding that SmileyLlama can combine its knowledge gained during single-objective optimization to perform well at a task specifying multiple objectives, elicited by combining the prompts (as opposed to requiring training on both prompts), which is a welcome outcome. Even so, there are still limitations to and trade-offs within the SmileyLlama framework and within our investigation for drug discovery. Additional factors for good drug candidates must also inhibit ‘off-target effects’ and/or be robust to mutation of the protein or virus among other downstream requirements. While SmileyLlama was not explicitly optimized for generating molecules with these qualities in this work, the DPO framework laid out here should be extensible to optimizing molecules for these characteristics. Even so, while DPO improves adherence to the prompt, it does so at the cost of narrowing the distribution of properties or diversity, which may not be desirable in all application areas or early stages of discovery. Furthermore, SmileyLlama still struggles in data-poor regimes, for example in the task of generating macrocycles.

The prompting and optimization framework for modifying LLMs to explore chemical space broadly or to narrow the search to specific regions shown here could also be leveraged for molecular design outside of drug discovery, such as the use of SMILES for elaborating on transition metal complexes^55^. One could also imagine that casting a chemical problem as a linguistic construct could enable other applications, such as our recent work on chemical synthesis^28^. As with many of the fields touched by LLMs this decade, the newly opened frontier of possibility in chemistry is as vast as it is exciting.

## Methods

### Details of properties for fine-tuning

#### Overview of selected properties for fine-tuning

When fine-tuning Llama to generate drug-like molecules, we carefully assess various design choices and proceed with the following properties, emphasizing those that medicinal chemists would consider when proposing de novo drug molecules. We categorized and summarized all 12 properties into 4 subgroups as follows.

- Physiochemical properties. Absorption, distribution, metabolism and excretion (ADME) are the crucial criteria to quantify the localization and concentration of drug molecules within the body after administration. As a result, we build on the list of properties proposed in the classical Lipinski’s rule-of-five^39^ with some modern additions such as TPSA to generate drug-like molecules that could demonstrate decent ADME.
  - Number of hydrogen bond donors (#HBD)
  - Number of hydrogen bond acceptors (#HBA)
  - MW
  - log of partition coefficient (log*P*)
  - TPSA
  - Fraction of *sp*^3^-hybridized carbon atoms (*Fsp*^3^)
- Structure flexibility features. Binding sites within a targeted biomolecule (most often a protein) display by nature complex 3D geometry, with key potential sites of drug–target interactions (amino acid side chains, as an example) somewhat fixed in space. The protein, however, has a dynamic structure, and even the binding pocket undergoes changes in shape. Drug-like molecules need to be sufficiently rigid to enable efficient interactions with their target protein, including, in most cases, a high degree of selectivity over corresponding interactions with related proteins. Perhaps less intuitive is that drug-like molecules must be flexible enough to maintain those interactions as the protein adapts its conformation. There is a ‘Goldilocks principle’ at play, where too rigid or too flexible are each undesired extremes. Here, we chose the following two properties to account for the flexibility aspect.
  - Number of rotatable bonds (#rot)
  - Whether the molecule contains a macrocycle (defined as an eight-membered ring or larger)
- Pattern-based features. In practical drug discovery, there are always some key patterns and/or scaffolds that medicinal chemists would like to hold onto or get rid of. For instance, in the lead optimization phase, retaining the key moiety and desired chemical formula are rather essential. Meanwhile, avoiding chemically unstable groups, PAINS molecules^56^ and molecules that would cause structure alerts could increase the chance of success in development. Therefore, we have the following three properties for fine-tuning.
  - Avoidance of undesirable chemical patterns
  - Retention of specified substructure (between 50 Da and 250 Da in MW)
  - Chemical formula
- Covalent warhead feature. Drugs can be broadly categorized into noncovalent and covalent drugs, depending on whether the drug reacts with its target. That is, an electrophilic group of a covalent inhibitor might form a bond with a nucleophilic amino acid side chain of its target protein. The reactive functional group of a covalent inhibitor is called a warhead. While most drugs are noncovalent, either can be desired. To give the model the ability to generate covalent binders, we also curated common covalent warhead-related SMARTS patterns from the Enamine fragment library^38^ to indicate whether our generated molecules have the capacity to covalently bind to the target or not.
  - Whether the molecule contains common covalent warhead-related SMARTS patterns, and which of these patterns appear in the molecule

#### Prompting options used in fine-tuning

To incorporate the properties mentioned above into the training, we used several ways of prompting to satisfy the requirement from target uses.

For numerical properties, including all physiochemical properties and #rotatable bonds, we prompted Llama by providing a specific range that the training molecules falls into for that specific category. All the cutoff values used for ranges are either commonly used standards in drug discovery or derived from the training distribution. Besides the range guidance, we added the prompt that tells Llama exactly how many #HBDs and #HBAs are contained in the training data, enabling more nuanced generation. If a property falls into multiple valid ranges—for instance, four H-bond donors satisfies all of 4, 5 and 7—we randomly select one of these ranges to include in the prompt (if the property is chosen to be included in the prompt). It is important that the set of ranges for a property spans all possible molecules; otherwise, a prompt that omits information may bias the model toward generating molecules with property values outside the defined ranges. If we never include information in the prompt about molecules with more than seven H-bond donors, but sometimes include the number of H-bond donors when it is seven or fewer, then omitting this information may bias the model toward assuming that the number of H-bond donors is greater than seven. Doing this during training would bias results during inference. This is the same reason we sometimes explicitly specify undesirable properties in the prompt, such as the presence of bad SMARTS patterns. If the random number generator decided that a prompt should contain a substructure but the SMILES in question did not have any BRICS substructures, we added ‘no BRICS substructure’ to the list of properties in lieu of a substructure.

For other categorical properties, we used a combination of RDKit modules, SMILES strings and SMILES arbitrary target specification (SMARTS) strings to recognize if certain properties or chemical patterns are present in the training input. Unlike the objective of containing the scaffold exactly, chemical pattern avoidance and covalent warhead recognition required matching of more general substructures and/or certain functional groups. Here, we used SMARTS strings as our representation because of its ability of matching chemical patterns. More details about the specific SMARTS patterns used are shown later in this section.

Below is a detailed list of possible components of that could appear in a training prompt.

- *N* H-bond donors, *N* = ≤3, ≤4, ≤5, ≤7, >7
- *N* H-bond acceptors, *N* = ≤3, ≤4, ≤5, ≤10, ≤15, >15
- *N* MW, *N* = ≤300, ≤400, ≤500, ≤600, >600
- *N* log*P*, *N* = ≤3, ≤4, ≤5, ≤6, >6
- *N* rotatable bonds, *N* = ≤7, ≤10, >10
- *N* fraction *sp*^3^, *N* = < 0.4, > 0.4, > 0.5, >0.6
- *N* TPSA, *N* = ≤90, ≤140, ≤200, >200
- a macrocycle, no macrocycles
- has bad SMARTS, lacks bad SMARTS
- has covalent warheads, lacks covalent warheads
- substructure of *a_smiles_string*
- a chemical formula of *formula*

#### SMART patterns used to identify bad chemical groups

Li et al. pointed out a list of bad chemical patterns that exists in ChEMBL database, which will negatively affect compound generation^14^. In this work, we used the same list of SMARTS patterns as their work to avoid bad patterns, including cyclopentadiene, cyclopentadiene ylidenes, aromaticity-breaking tautomers, antiaromatic system, unstable halogen–heteroatom bonds, unstable fused rings, allenic system, thiazyl linkages and peroxide bonds. In Supplementary Table 2, we also present the frequency of sampling undesirable chemical groups in ChEMBL and across different generative models.

- [C^2]1=[C^2]-[C^2]=[C^2] ~ [C;!d4] ~ [C;!^2;d2]1
- [C^2]1 ~ [C^2] ~ [C^2] ~ [C^2] ~ [C;!^2;d2] ~ [N]1
- [#6^2]1 ~ [#6^2] ~ [#6^3;!d4] ~ [#6^2]2 ~ [#6^2] ~ [#6^2] ~ [#6^2] ~ [#6^2](~ [*]) ~ [#6^2] ~ 2 ~ [#6^2] ~ 1
- [#6]1(=[*])[#6]=[#6][#6]=[#6]1
- [#6]1=[#6][R2-]=[R2-]1
- [#6^2]1 ~ [#6^2] ~ [#6^2] ~ [#6^2] ~ [#6^1] ~ [#6^1] ~ 1
- [#7,#8,#16]-[#9,#17,#35,#53]
- [r3,r4]@[r5,r6]
- [*]=[#6,#7,#8]=[*]
- [#7,#16]=[#16]
- [#8]-[#8]

In addition to the patterns mentioned above, we use the following SMARTS patterns to enforce our generated pyrroles to be one of the following correct forms.

- [N^2]1 ~ [C,N;^2](=[*]) ~ [C,N;^2] ~ [C,N;^2] ~ [C^3]1
- [N^2]1 ~ [C,N;^2] ~ [C,N;^2](=[*]) ~ [C,N;^2] ~ [C;^3]1
- [N^2]1 ~ [C,N;^2] ~ [C,N;^2] ~ [C,N;^2](=[*]) ~ [C;^3]1
- [C,N;^2](=[*])1 ~ [N;^2] ~ [C,N;^2] ~ [C,N;^2] ~ [C;^3]1
- [C,N;^2]1 ~ [N;^2] ~ [C,N;^2](=[*]) ~ [C,N;^2] ~ [C;^3]1
- [C,N;^2]1 ~ [N;^2] ~ [C,N;^2] ~ [C,N;^2](=[*]) ~ [C;^3]1

#### SMART patterns used to encode common covalent warhead-related functional groups

Common covalent warheads are extracted from the Enamine Covalent Screening and Covalent Fragment Library^38^. The list of SMARTS strings is shown below.

- sulfonyl fluorides: [#16](=[#8])(=[#8])-[#9]
- chloroacetamides: [#8]=[#6](-[#6]-[#17])-[#7]
- cyanoacrylamides: [#7]-[#6](=[#8])-[#6](-[#6]#[#7])=[#6]
- epoxides: [#6]1-[#6]-[#8]-1
- aziridines: [#6]1-[#6]-[#7]-1
- disulfides: [#16]-[#16]
- aldehydes: [#6](=[#8])-[#1]
- vinyl sulfones: [#6]=[#6]-[#16](=[#8])(=[#8])-[#7]
- boronic acids/esters: [#6]-[#5](-[#8])-[#8]
- acrylamides: [#6]=[#6]-[#6](=[#8])-[#7]
- cyanamides: [#6]-[#7](-[#6]#[#7])-[#6]
- chloroFluoroAcetamides: [#7]-[#6](=[#8])-[#6](-[#9])-[#17]
- butynamides: [#6]#[#6]-[#6](=[#8])-[#7]-[#6]
- chloropropionamides: [#7]-[#6](=[#8])-[#6](-[#6])-[#17]
- fluorosulfates: [#8]=[#16](=[#8])(-[#9])-[#8]
- beta lactams: [#7]1-[#6]-[#6]-[#6]-1=[#8]

To assess SmileyLlama’s performance on generating molecules with specified properties, as in Table 2, we investigate SmileyLlama’s performance on the following 387 tasks, grouped into the families for which the averages are shown in the table.

- exactly *k* H-bond donors, from *k* = 0 to *k* = 5
- exactly *k* H-bond acceptors, from *k* = 0 to *k* = 10
- ≤*k* H-bond donors, for *k* = 3, 4, 5, 7
- ≤*k* H-bond acceptors, for *k* = 3, 4, 5, 10, 15
- ≤*k* MW, for *k* = 300, 400, 500, 600
- ≤*k* log*P*, for *k* = 3, 4, 5, 6
- ≤7, ≤10, >10 rotatable bonds
- >0.4, >0.5, >0.6, <0.4 fraction *sp*^3^
- ≤90, ≤140, ≤200 TPSA
- a macrocycle
- no macrocycles
- has bad SMARTS (not shown in table but included for completeness)
- lacks bad SMARTS
- lacks covalent warheads
- has covalent warheads (one for each of the 16 covalent warheads in the section above)
- a substructure of (one of each of 320 Enamine fragments^38^)
- < = 5 H-bond donors, < = 10 H-bond acceptors, < = 500 Molecular weight, < = 5 LogP
- < = 3 H-bond donors, < = 3 H-bond acceptors, < = 300 Molecular weight, < = 3 LogP

### Prompt formats and examples

We assess the ability of Llama to generate SMILES strings as a baseline. Below are examples of system and user prompts to illustrate the methods we used to prompt Llama and SmileyLlama. The Llama prompts are constructed using the Llama instruction-tuning format, while the SmileyLlama, robotic prompt, and blank prompts use the Alpaca format to reproduce the setup used in the most recent supervised fine-tuning of the foundation model.

For the case of Llama zero-shot, we use the following format, with no prefilled responses, when generating data for the GuacaMol benchmark. We chose to use a user prompt asking for ‘no other output’ because, in our informal experiments, Llama would often respond indirectly, including English text discussing SMILES strings without this explicit instruction to generate only SMILES strings.


System prompt:


‘You love and excel at generating SMILES strings of drug-like molecules’


User prompt:


‘Please generate a drug-like smiles string and no other output:’

Llama-k-shot has *k* prefilled responses using ChEMBL molecules. In this example, we will show the system prompt and user prompt with three prefilled ChEMBL molecules.:


System prompt:


‘You love and excel at generating SMILES strings of drug-like molecules’


User prompt:


‘Please generate a drug-like smiles string and no other output:’


Response:


‘Cc1cc(c(C)n1CCOC)C(=O)CSc1nc2nc(cc(n2n1)C)C’


User prompt:


‘Please generate a drug-like smiles string and no other output:’


Response:


‘N(c1nc([C@]23N=C(N)SC[C@@H]3C[C@H](C)OC2)sc1)C(c1ncc(nc1)OC)=O’


User prompt:


‘Please generate a drug-like smiles string and no other output:’


Response:


‘c1nn([C@H](C(NCCc2sccc2C)=O)CC)cc1’


User prompt:


‘Please generate a drug-like smiles string and no other output:’

Moving on to prompts used for supervised fine-tuning and subsequent inference, we first give system and user prompts for SmileyLlama. Because the user prompt is dependent on whether any properties are selected to be specified, we give both versions here. The user prompt with no properties should sample from a distribution most similat to ChEMBL, so we use this format when sampling SMILES for assessment of the GuacaMol benchmark.


System prompt:


‘You love and excel at generating SMILES strings of drug-like molecules’

User prompt (no properties selected):

‘Output a SMILES string for a drug-like molecule:’

User prompt (properties selected):

‘Output a SMILES string for a drug-like molecule with the following properties: <property 1>, <property 2>, <property 3>, …’

Below are the system and user prompt used in the ‘robotic prompt’ control of prompt phrasing for GuacaMol inference. It should be noted that the SFT dataset for this ‘robotic prompt’ control had the same user prompts as SmileyLlama (including specified properties), but a system prompt of ‘Generate a SMILES string of a drug-like molecule according to the user’s input’:


System prompt:


‘Generate a SMILES string of a drug-like molecule according to the user’s input:’

User prompt (no properties selected):

‘Output a SMILES string for a drug-like molecule:’

User prompt (properties selected):

‘Output a SMILES string for a drug-like molecule with the following properties: <property 1>, <property 2>, <property 3>, …’

Below are the system an user prompts for the ‘Blank prompt’ example in Table 1.


System prompt:


‘’

User prompt (no properties selected):

‘’

User prompt (properties selected):

‘<property 1>, <property 2>, <property 3>, …’

### Additional training details

We performed both SFT and DPO on Llama using the Axolotl Package^57^. For both SFT and DPO, we use the low-rank adaptation (LoRA) applied to the linear layers of the model and FlashAttention with an Adam optimizer, cross-entropy loss and a cosine learning rate scheduler with a maximum learning rate of 2 × 10^−4^ for SFT and 2 × 10^−5^ for DPO^58,59^. All prompts were formatted according to the Alpaca instruction format^60^. Additional parameters for our training are a LoRA rank of 32, a LoRA alpha of 16, a LoRA dropout of 5% and 10 warmup steps. We inherit these hyperparameters from standard practice with Axolotl, such as LoRA hyperparameters identical to these used in the Hermes 3 SFT^61^. For SFT, we trained for 1 epoch using a batch size of 64 on a single 4xA40 node for approximately 32 h with a validation set ~5% the size of the original, an amount that would cost approximately US$53 in October 2025 on Vast.ai. We also note that we randomized the SMILES string representation of each molecule, and we tokenized all SMILES strings with the Llama3 tokenizer^17^ when interfacing with SmileyLlama.

We used the HuggingFace Transformers^62^ library to perform inference. Unless otherwise indicated, we used a temperature of 1.0. To avoid biasing generations, we do not restrict the possible tokens produced at any particular step by setting top_p or top_k. We allow a maximum of 128 new tokens, which truncates the size of the generated SMILES strings, noting that larger values of this hyperparameter lead to generally similar results, with the exception of a broader, less drug-like distribution of molecules after several iterations of optimization for MPro binding within the iMiner framework. We also note that SmileyLlama can fall victim to the repeat curse; on occasion, SmileyLlama will continue producing tokens indefinitely with some repetitive pattern on sufficiently long molecules, emphasizing the need for a token cutoff.

### GuacaMol benchmark definitions

The GuacaMol benchmark assesses generative models based on five metrics^34^.

- Validity: the proportion of the first *N* generated strings that are RDKit-parsable and have more than 0 atoms.
- Uniqueness: the number of distinct molecules in a set of *N* total valid generated strings, divided by *N*.
- Novelty: the number of *N* valid, unique generated strings that do not represent a molecule in the training set.
- KL divergence: the distribution of a variety of physiochemical descriptors is calculated for both the generated molecules and the training set, and their similarity is assessed through KL divergence.
- Fréchet ChemNet distance: the Fréchet distance between the distributions of activations of generated molecules and those of the training set, computed on the penultimate layer of a neural network called ChemNet.

All benchmarks were performed with 10,000 samples at a temperature *T* = 1.0 and a maximum of 128 new tokens for Llama and SmileyLlama. We note that, due to the proprietary nature of Llama’s training data and the SMILES contained therein, our assessments of Llama zero-shot’s novelty with respect to ChEMBL is not as meaningful as the novelty assessment of SmileyLlama and the CLMs. Finally, we note that FCD_Guac_ = exp(−0.2 × FCD) compresses the FCD distances themselves for the convenience of defining a 0 to 1 value like the other GuacaMol metrics^34^. Hence, the scores should actually be considered from a log perspective such that an FCD below 5 (that is, FCD_Guac_ of 0.37) is in strong agreement with distributions of drug-like properties. Only when the FCD_Guac_ score decreases by one to two orders of magnitude are scores considered poor. We recommend that future work report the more straightforward FCD distances themselves to avoid this interpretative confusion.

### iMiner reinforcement learning with SmileyLlama

The iMiner generative model uses an Average Stochastic Gradient Descent Weight-Dropped Long Short-Term Memory (AWD-LSTM)^14^ recurrent neural network that predicts the probability of string tokens, which are concatenated onto a molecular string representation until a complete molecule is generated. In the subsequent RL stage, 2,000 molecules in each epoch (typically ~50 epochs) are sent to AutoDock simulations in parallel, and the docking scores are circled back to the RL model to adjust its parameters so that molecules generated in the next iteration will have better scores while retaining drug likeness. Given the ascendancy of attention mechanisms and transformer architectures, such as those inherent in SmileyLlama, we replaced the generative AWD-LSTM component of iMiner with SmileyLlama, and replaced the iMiner optimization algorithm, proximal policy optimization (PPO)^14^ with DPO. This is largely due to the high memory requirement of PPO; because DPO does not need to fit a reward model to the predicted and realized rewards, it requires far less memory than PPO. This becomes even more true when tuning a large model compared with a small one, because generally the reward model trained is the same size as the language model used to generate strings.

We use a scoring function of three times the docking score plus the iMiner drug-likeness function^14^ to score all molecules per iteration. The drug-likeness score is an extension of the widely used quantitative estimate of drug-likeness (QED)^14^, and is defined as

1 $${S}_{\mathrm{DL}}(X)=\mathop{\sum }\limits_{i}{\sigma }_{i}\log {p}_{i}({\mathrm{prop}}_{i}(X)),$$

where prop_*i*_(*X*) calculates the *i*th property of a molecule *X*, and *p*_*i*_ is defined by the probability distribution of property *i* by all molecules in the ChEMBL database. The parameter *σ*_*i*_ is defined as

2 $${\sigma }_{i}={S}_{i}^{-1}/\mathop{\sum }\limits_{j}{S}_{j}^{-1},$$

where *S*_*i*_ is the entropy of the distribution of property *i*,

3 $${S}_{i}=-\mathop{\sum }\limits_{x}{p}_{i}(x)\log {p}_{i}(x),$$

such that a narrower distribution from the ChEMBL database contributes more to the drug likeliness score, and defines the weights for each property as proportional to the inverse of the entropy. Invalid molecules were assigned a score of −10. More details can be found in the original iMiner work^14^.

After every 2,000 distinct molecules are generated per iteration using SmileyLlama, we score each molecule and randomly pair it with another for the DPO procedure. The molecule with the higher score is designated as the ‘winner’ and the other as the ‘loser’, and we construct a dataset with eight such pairings for each molecule and then optimize SmileyLlama using DPO with respect to this dataset. We empirically found eight pairs to work well; more than eight pairs tended to lead to a lack of diversity, whereas fewer than eight pairs resulted in less sample efficient optimization. This process is described in Supplementary Algorithm 3.

### SmileyLlama outside of chemical language modeling

While SFT clearly alters the model, one might wonder whether SmileyLlama retains LLM abilities unrelated to exploring chemical space. We find that SmileyLlama inherits some ability to process natural language; however, SmileyLlama will sometimes respond to chemistry-related queries with a SMILES string rather than a sensible, natural-language answer. Below are four sample responses from SmileyLlama to four prompts. Each was generated with greedy decoding, which is equivalent to sampling at a temperature of 0. All responses were generated using the Alpaca instruction format, with a system prompt/instruction of ‘You are a helpful assistant’. As shown below, SmileyLlama is capable of writing working Python code for simple functions and answering some questions in natural language. This demonstrates that, while SmileyLlama was designed to be a CLM, it still inherits some of Llama’s natural language processing ability. However, this is not perfect, as SmileyLlama will sometimes respond to chemistry-related queries with a SMILES string, rather than the correct answer.


Input: What is the molecular weight of hydrazine?


Output: ‘N1N=C(C)C(C1)C’

(Note: this is the SMILES representation of 3,4-dimethyl 2-pyrazoline, rather than hydrazine)


Input: What is a Diels-Alder reaction in organic chemistry?


Output: ‘A Diels-Alder reaction is a [4+2] cycloaddition between a diene and a dienophile.’


Input: Who was Christopher A. Lipinski in the field of chemistry? What was his ‘Rule’?.


Output: ‘Christopher A. Lipinski was a chemist who developed the ‘Lipinski’s Rule of Five’ which states that for a drug to be orally bioavailable, it should have the following properties:

1. C(C)Cn1c2c(cccc2)c(c1)C(Nc1ccccc1)=O
2. A molecular weight of 500 or less
3. No more than 5 H-bond donors
4. No more than 10 H-bond acceptors’

An example of code generation is given in Supplementary Fig. 8. Furthermore, we used the Language Model Evaluation Harness to test SmileyLlama’s performance on the MMLU GPQA, Math-Hard, wikitext and MMLU-Pro benchmarks^49,51–53^. The results in Supplementary Table 3 and Supplementary Fig. 7 show that SmileyLlama generally performs only a little worse on most tasks compared with Llama. For practical purposes, the SFT procedure for creating SmileyLlama seems to have created additional functionality to Llama, and we expect that much larger LLMs will be even better at sustaining their original capabilities.

#### Unphysical requests

Because SmileyLlama can take in prompts purely in natural language, it is possible to give SmileyLlama a nonsensical instruction. Here, we ask SmileyLlama to generate molecules that are impossible, in principle, to generate. In both cases we investigate, SmileyLlama does not refuse the request. Rather, it will generate SMILES strings not satisfying the (impossible) conditions.

First, we ask SmileyLlama to generate a molecule that has two properties in contradiction with each other: a substructure of 1,2,3,4-tetramethoxybenzene and three or fewer H-bond acceptors. This is impossible because 1,2,3,4-tetramethoxybenzene has four H-bond acceptors itself, and so any molecule with this as a substructure must have at least that many H-bond acceptors. With greedy decoding, SmileyLlama gives the following molecule:


Input: Output a SMILES string for a drug like molecule with the followingproperties: a substructure of c1c(OC)c(OC)c(OC)c(OC)c1, < = 3 H-bond acceptors:


Output: ‘c1c(c(c(c(c1)OC)OC)OC)C1C2C(CCC=1)C1C(CCC=2)C1’

This molecule has a substructure of 1,2,3-trimethoxybenzene, which is similar but not identical to 1,2,3,4-tetramethoxybenzene. However, the output molecule has three H-bond acceptors, which obeys the second instruction in the prompt.

We can also ask for a blatantly unphysical molecule, such as one with negative six H-bond donors:


Input: Output a SMILES string for a drug like molecule with the following properties: < = -6 H-bond donors:


Output: ‘c1c2c(ccc1)C(=O)N(C2=O)CC(Nc1ccc(cc1)C(=O)Nc1ccc(cc1)C(Nc1ccc(cc1)

C(Nc1ccc(cc1)C(Nc1ccc(cc1)C(Nc1ccc(cc1)C(Nc1ccc(cc1)C(Nc1ccc(cc1)

C(Nc1ccc(cc1)C(Nc1ccc(cc1)C(Nc1ccc(cc1)C(Nc1ccc(cc1)C(Nc1ccc(cc1)C(N’

This cut off at 128 new tokens, which was the maximum for this particular experiment. To our knowledge, it will repeat this pattern indefinitely, which we have tested for up to 8,192 new tokens, a pattern not new to LLMs.

This demonstration of SmileyLlama’s behavior is not exhaustive; there are probably many other requests that elicit responses differing from what a naive extrapolation of these anecdotes would suggest. Here, we simply aimed to provide examples of some of the interesting behaviors SmileyLlama can exhibit when asked to perform tasks outside the drug discovery domain for which it was originally developed.

### Reporting summary

Further information on research design is available in the Nature Portfolio Reporting Summary linked to this article.

## Supplementary information

Supplementary Information Supplementary Tables 1–3 and Figs. 1–8.

Reporting Summary

Supplementary Data SMILES strings and scores for selected generated molecules. All SA scores were calculated using the method by Ertl and Schuffenhauer^48^.

## Source data

Source Data Fig. 1 Statistical source data for Fig. 1.

Source Data Fig. 2 Statistical source data for Fig. 2.

Source Data Fig. 3 Statistical source data for Fig. 3.

## Data Availability

The data included here contain all SMILES strings we used from ChEMBL (chembl.txt), the dataset containing the BRICS substructures of these ChEMBL molecules, the dataset containing prompts and responses used for SFT to create SmileyLlama from Llama, and the dataset used to create SmileyLlama-DPO-opt from SmileyLlama. Source data for all figures is available with this manuscript. Data used for this study are available via GitHub at https://github.com/THGLab/SmileyLlama (ref. ^63^) and via figshare at 10.6084/m9.figshare.30854573 (ref. ^64^). Source data are provided with this paper.

## Code availability

Code used for this study is available via GitHub at https://github.com/THGLab/SmileyLlama (ref. ^65^), and models can be found in refs. ^66–68^.
