# Supplementary material for: SmileyLlama: modifying large language models for directed chemical space exploration
Source: Nat Comput Sci. 2026 May 11;6(8):856–69. doi: 10.1038/s43588-026-00986-y (PMC13489950; doi:10.1038/s43588-026-00986-y)
Supplement: Supplementary file 1 [file 43588_2026_986_MOESM1_ESM.pdf]

nature computational science

Supplementary information

https://doi.org/10.1038/s43588-026-00986-y

SmileyLlama: modifying large language models for directed chemical space exploration

In the format provided by the authors and unedited

Table of Contents

Algorithms used for SFT and DPO..........2
Supplementary Tables 1-3..........3
Supplementary Figures 1 and 2..........4
Supplementary Figure 3..........5
Supplementary Figure 4..........6
Supplementary Figure 5..........7
Supplementary Figure 6..........8
Supplementary Figure 7..........9
Supplementary Figure 8..........10

1 Algorithms used for SFT and DPO

Algorithm 1 Pseudocode for generating the SFT dataset

```
for all SMILES_STRING ∈ ChEMBL do
    PROMPT ← "Output a SMILES string for a drug like molecule with the following properties:"
    for all (PROPERTY_FUNCTION, PROPERTY_NAME) ∈ shuffle(PROPERTIES) do
        if random_coin_flip() = HEADS then
            VALUE ← PROPERTY_FUNCTION(SMILES_STRING)
            VALUE_RANGE = RANDOM_RANGE_VALUE_FALLS_IN(VALUE)
            PROMPT ← PROMPT + PROPERTY_NAME + " of " + VALUE_RANGE + ", "
        end if
    end for
    prompts.append(PROMPT)
    completions.append(SMILES_STRING)
end for
```

Algorithm 2 Pseudocode for generating the DPO dataset for improving prompt obedience

```
for all (SMILES_STRING, CONDITIONS) ∈ RESPONSES do
    if MEETS_CONDITIONS(SMILES_STRING, CONDITIONS) = True then
        winners.append(SMILES_STRING)
    else
        losers.append(SMILES_STRING)
    end if
end for
pairs ← randomly_pair(winners, losers)
assert: |pairs| = min{|winners|, |losers|}
```

Algorithm 3 Pseudocode for generating the DPO dataset for Mpro optimization every iteration

```
PROMPT ← "Output a SMILES string for a drug like molecule with the following properties: High SARS2PRO"
while |SMILES_LIST| < num_responses do
    SMILES_LIST ← SmileyLlama.inference(PROMPT, num_responses)
    SMILES_LIST ← SMILES_LIST.remove_redundancies()
end while
for SMILES ∈ SMILES_LIST do
    iMiner.compute_score(SMILES)
end for
for i = 1 to 8 do
    for SMILES ∈ SMILES_LIST do
        while True do
            RANDOM_OTHER_SMILES ← pick_random(SMILES_LIST)
            if iMiner.retrieve_score(SMILES) > iMiner.retrieve_score(RANDOM_OTHER_SMILES) then
                DATASET.append((PROMPT, SMILES, RANDOM_OTHER_SMILES))
                break
            else if iMiner.retrieve_score(SMILES) < iMiner.retrieve_score(RANDOM_OTHER_SMILES) then
                DATASET.append((PROMPT, RANDOM_OTHER_SMILES, SMILES))
                break
            end if
        end while
    end for
end for
```

2 Supporting Tables

Supplementary Table 1: *GuacaMol benchmarks comparing SmileyLlama to related models, ablations, and a model trained with a different prompt format.* Similar to Table 1 in the main text, we generate molecules at a temperature T =1.0 and a maximum of 128 new tokens. These benchmark scores suggest that the SmileyLlama SFT procedure outlined here can be extended towards adapting other LLMs into CLMs, including Qwen and smaller variants within the Llama 3 Herd. We also note that changing the prompt templates, as shown in the "robotic phrasing" and "blank prompt" rows, does not appear to significantly affect these results.

**Benchmark**	**Validity**	**Uniqueness**	**Novelty**	**KLdiv**$_{Guac}$	**FCD**$_{Guac}$
**SmileyLlama**	0.958	1.000	0.987	0.967	0.686
**robotic phrasing**	0.952	1.000	0.988	0.963	0.696
**blank prompt**	0.953	1.000	0.984	0.966	0.676
**SmileyLlama-1B**	0.911	1.000	0.987	0.968	0.588
**SmileyLlama-3B**	0.929	1.000	0.989	0.973	0.617
**SmileyQwen2.5-7B**	0.964	1.000	0.987	0.908	0.516

Supplementary Table 2: *Frequency of generating or sampling undesirable chemical structures in ChEMBL, SmileyLlama, and other SOTA methods.* Here, we randomly sample 10,000 molecules from the ChEMBL dataset, SmileyLlama and our retrained models to compare their statistics of five different categories of undesirable chemical patterns.

**Model**	ChEMBL	SmileyLlama	LSTM	GPT	S4
Unsaturated Benzene	0.55%	0.1 %	0.04%	0.04%	0.03%
Unsaturated Naphthalene	0.02%	0.01%	0.02%	0.01%	0.01%
Wrong Pyrrole	0.29%	0.02%	0.02%	0.03%	0.04%
Cyclopentadiene Ylidene	0.96%	0.1 %	0.04%	0.06%	0.05%
Benzyne	0.01%	0.0 %	0.0%	0.0%	0.0%

Supplementary Table 3: *Performance of Meta-Llama-3.1-8B-Instruct versus SmileyLlama in a variety of LLM benchmarks.* We report the mean score, assessed with the exact_match scoring method for all tasks besides perplexity.

**Model**	Llama-3.1-8B-Instruct	SmileyLlama
Wikitext (word perplexity) (↓)	8.6401	8.8934
MMLU (0-shot) (↑)	0.68	0.65
GPQA (chain of thought) (↑)	0.30	0.038
Math-Hard (↑)	0.24	0.039
MMLU-Pro (↑)	0.47	0.063

3 Supporting Figures

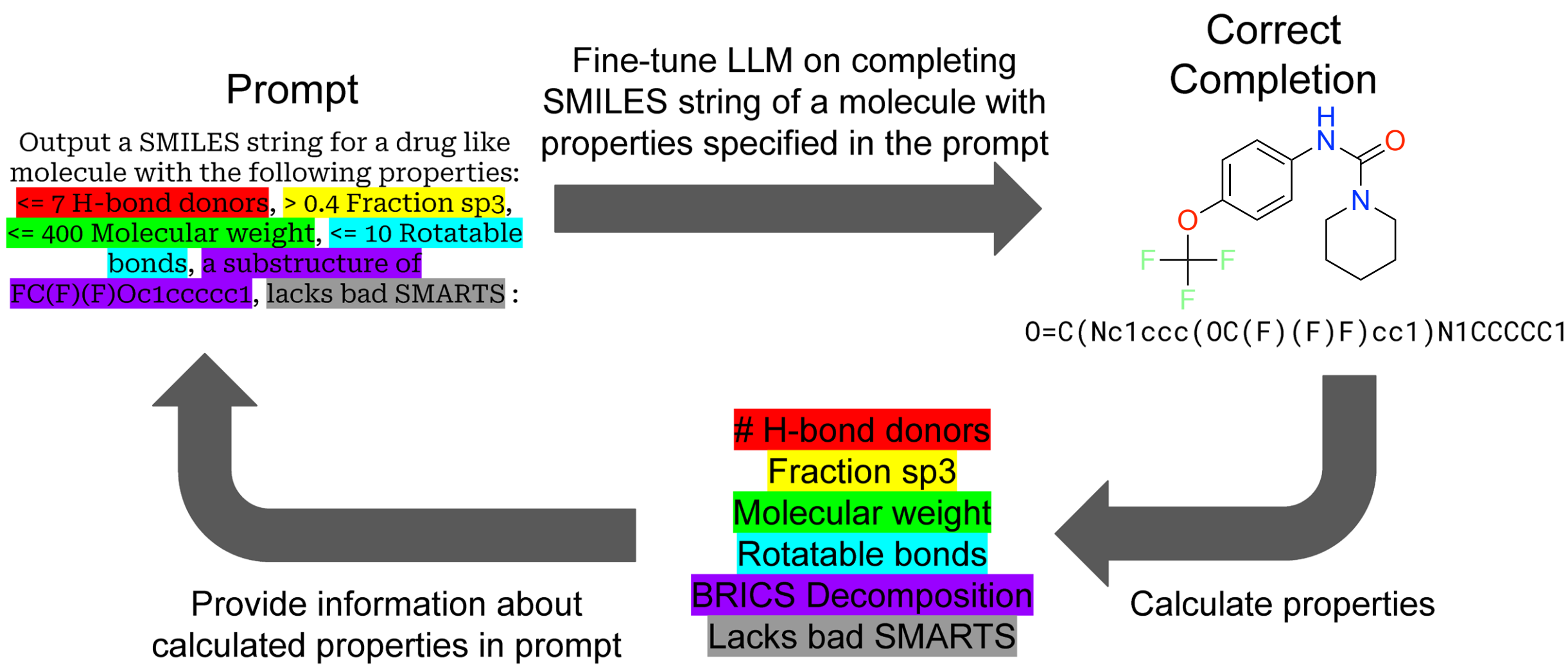

Supplementary Figure 1: *A visualization of the SFT workflow for Smiley-Llama.* Given the Llama-3.1-8B-Instruct model[1], we used prompt-response pairs consisting of calculated molecular properties and completed SMILES strings to fine-tune Llama on SMILES strings completions, yielding SmileyLlama. Crucially, we construct the prompt for each example using properties calculated from the correct response (a SMILES string from ChEMBLv33).

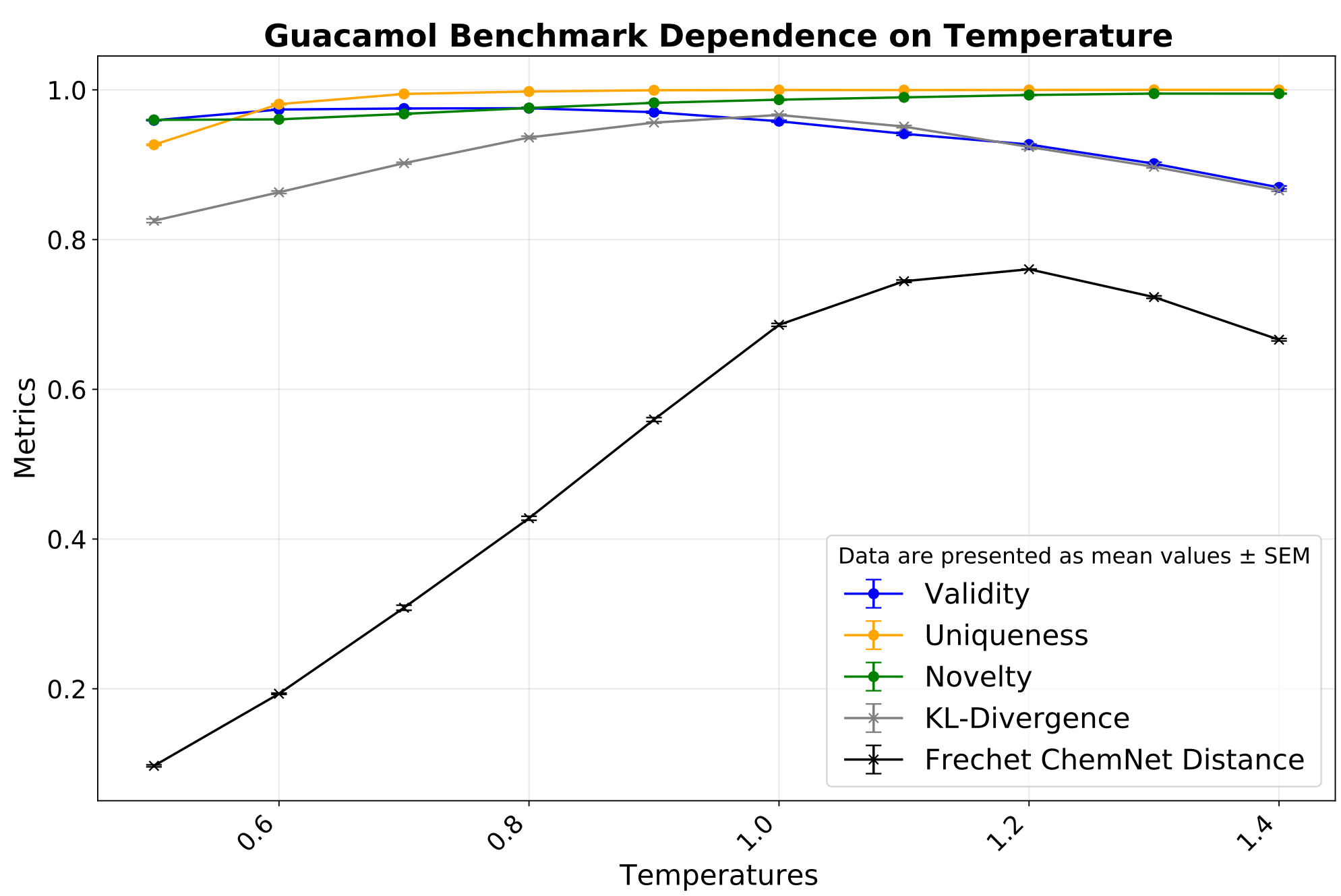

Supplementary Figure 2: *Guacamol benchmark results as a function of temperature.* We used n=3 runs to assess how validity, uniqueness and novelty depends on temperature, a parameter which affects the randomness of outputs generated during inference. Error bars are defined by the standard error. Temperature can be increased during inference for more random molecules, sacrificing validity for diversity and novelty. We generally use a temperature of T=1.0 during this study.

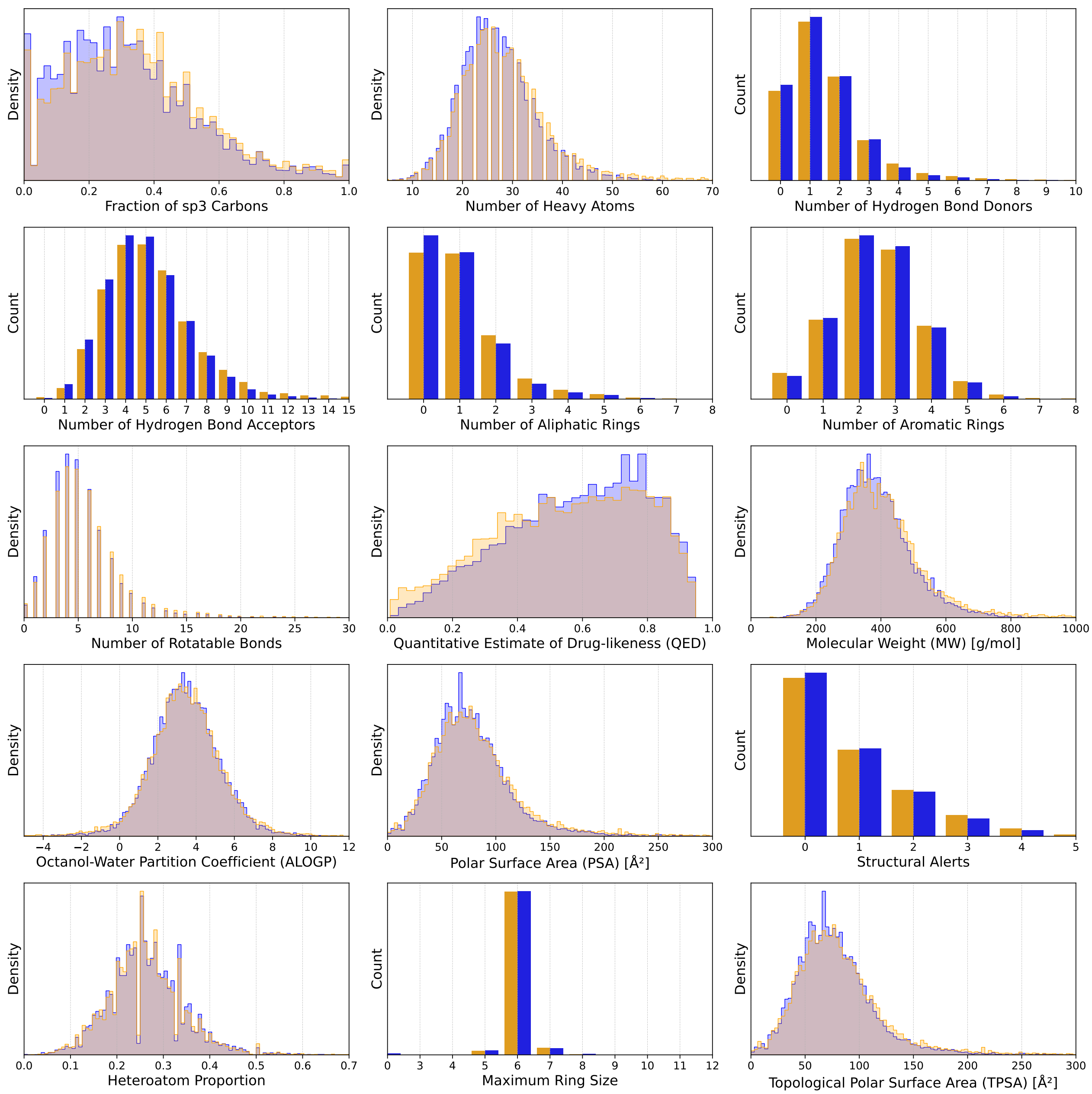

Supplementary Figure 3: *Distribution comparisons for different properties of the generated molecules from the LSTM CLM (blue) with molecules from the training dataset from ChEMBL (gold).* To be compared with Figure 1b in the main text and Figure S4.

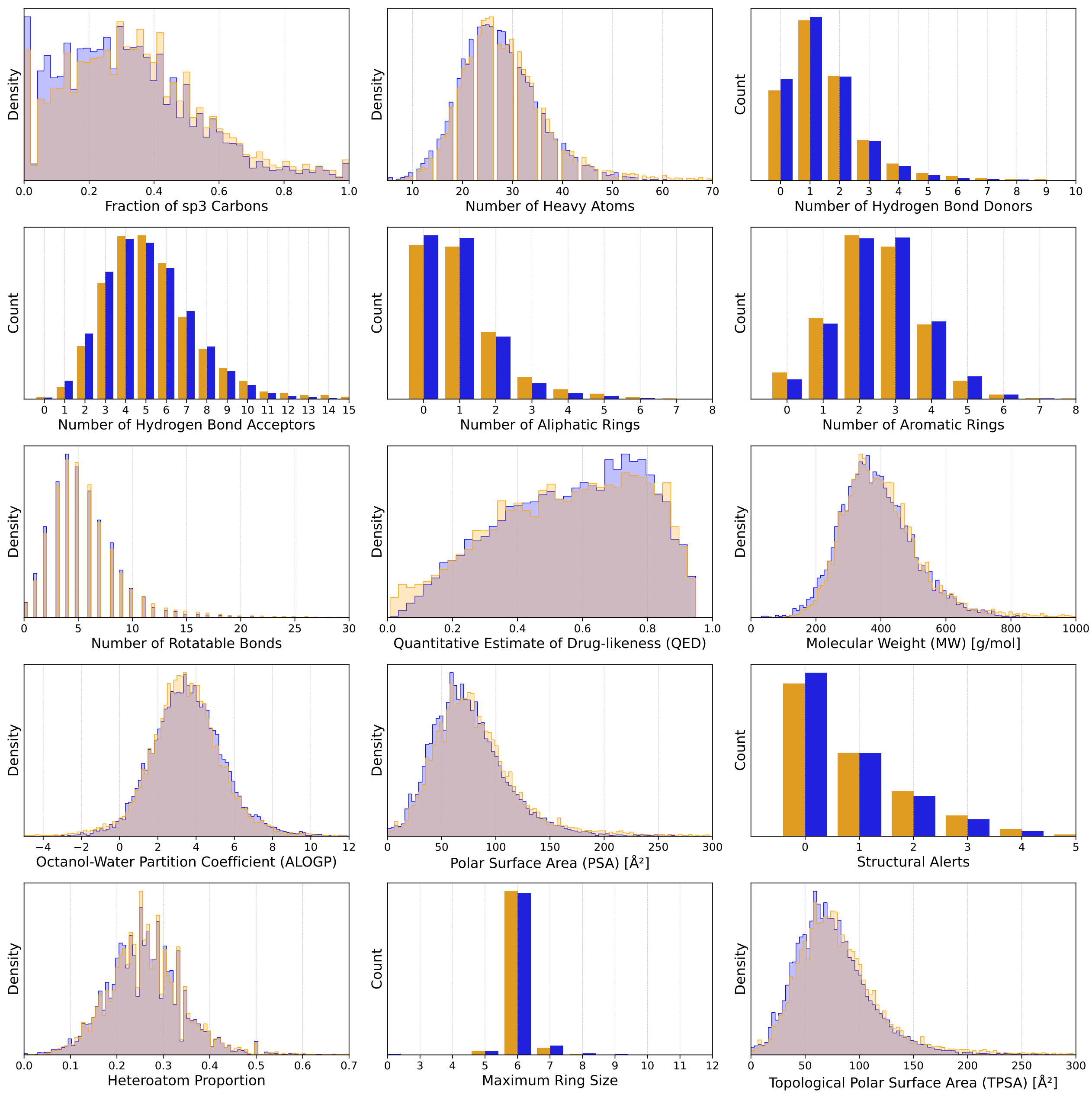

Supplementary Figure 4: *Distribution comparisons for different properties of the generated molecules from the GPT model (blue) with molecules from the training dataset from ChEMBL (gold).* To be compared with Figure 1b in the main text and Figure S3.

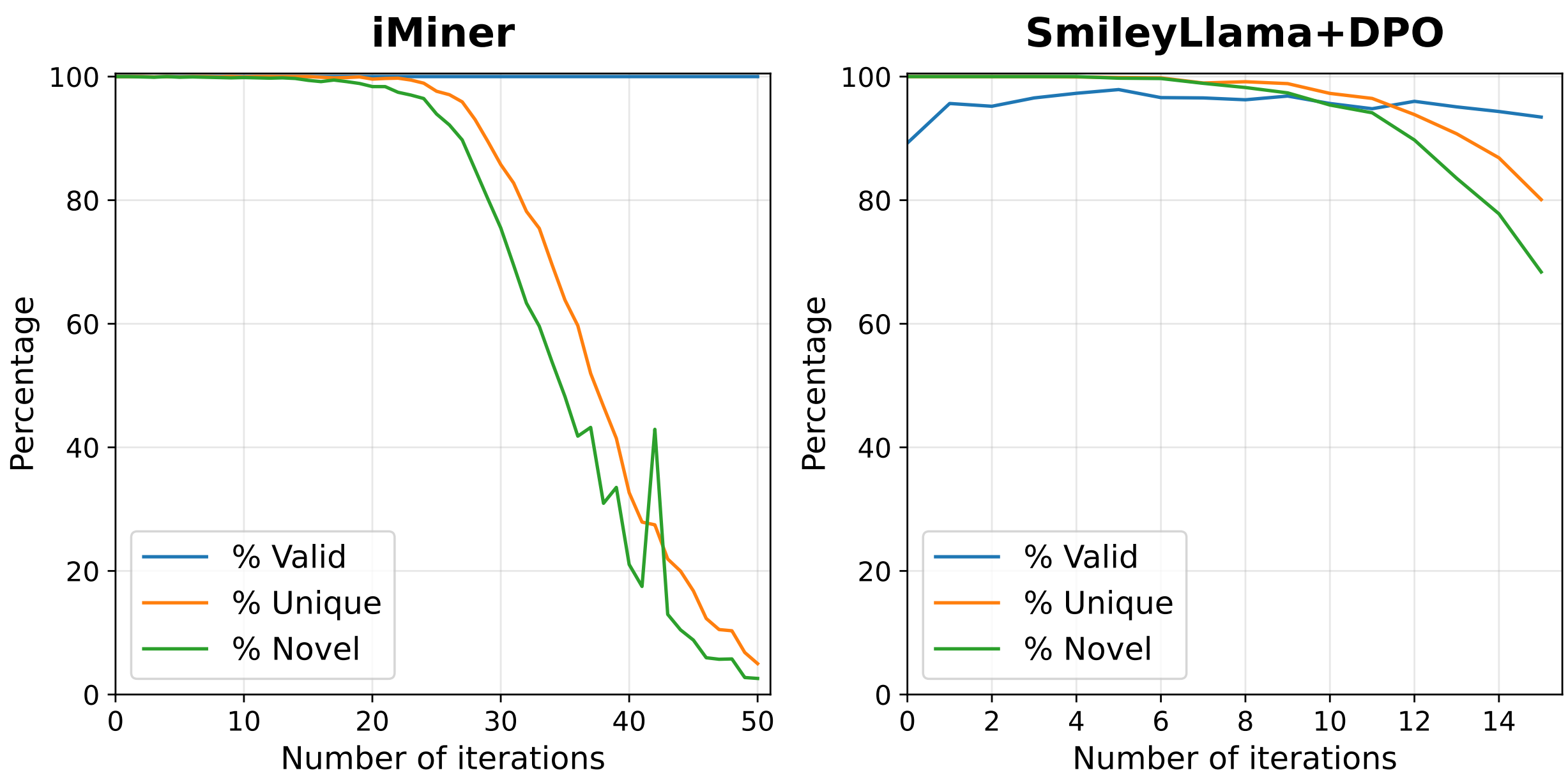

Supplementary Figure 5: *Validity, uniqueness and novelty over optimization for SARS2-Mpro inhibition* We plot the validity (fraction of molecules which are valid), uniqueness (number of distinct molecules) and novelty (fraction of molecules which have not been generated before) of molecules sampled from the generative models of iMiner with 2000 molecules per epoch, and for SmileyLlama with DPO (SL+DPO) with 2000 and 1000 molecules per epoch. Note that novelty is not defined with respect to ChEMBL; here it's a measure of how good the model is at generating molecules that it has not generated in a previous iteration.

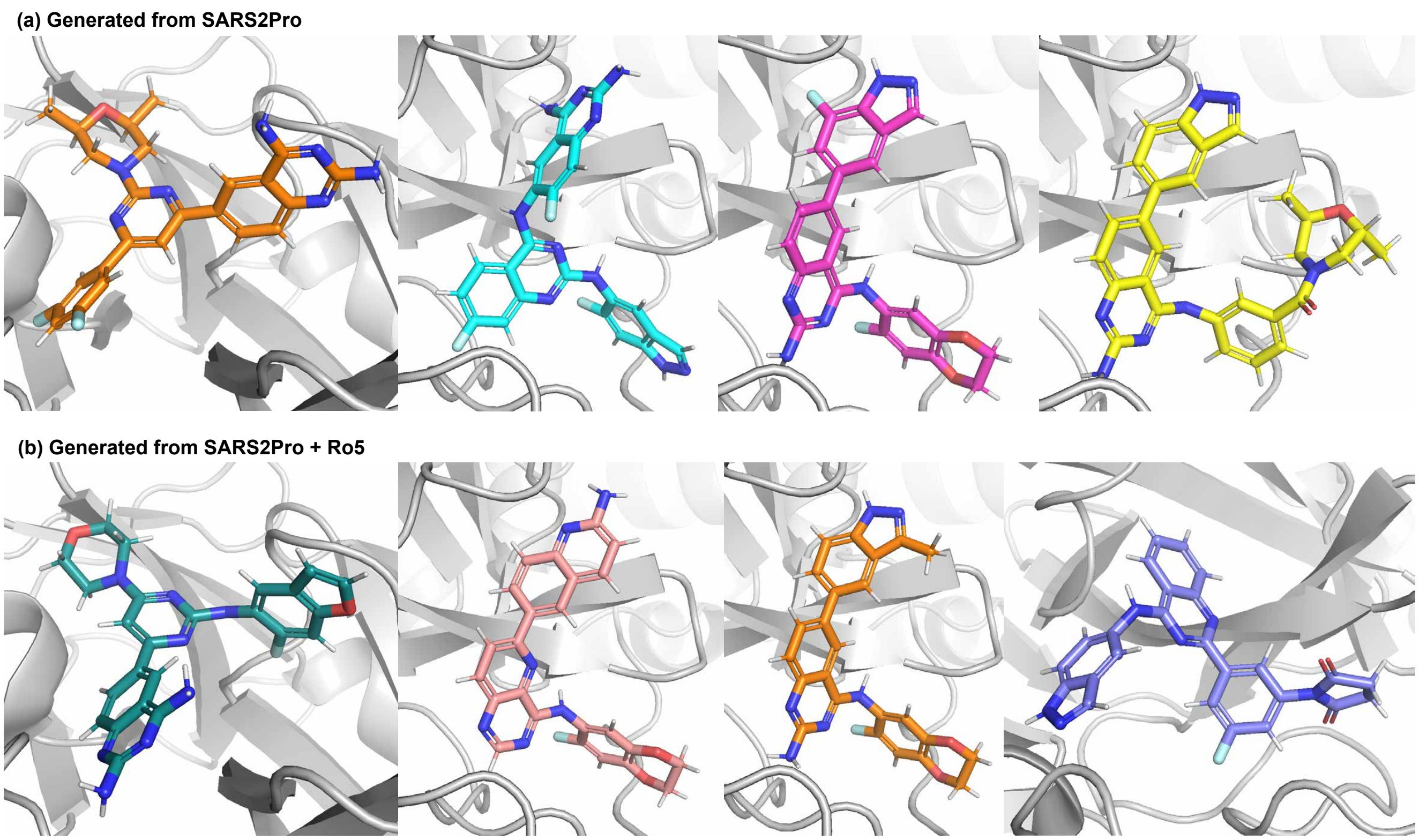

Supplementary Figure 6: *SmileyLlama de novo generated molecules in the active site of SARS2 main protease.* Surface rendering of the SmileyLlama generated molecules in the SARS2 Mpro canonical binding pocket. Generated by SmileyLlama after optimization with the **SARS2PRO** and **SARS2Pro+Ro5** prompts. Supplementary Table S4 provides their SMILES string and docking scores. Blue is nitrogen, white is hydrogen, red is oxygen, otherwise carbon

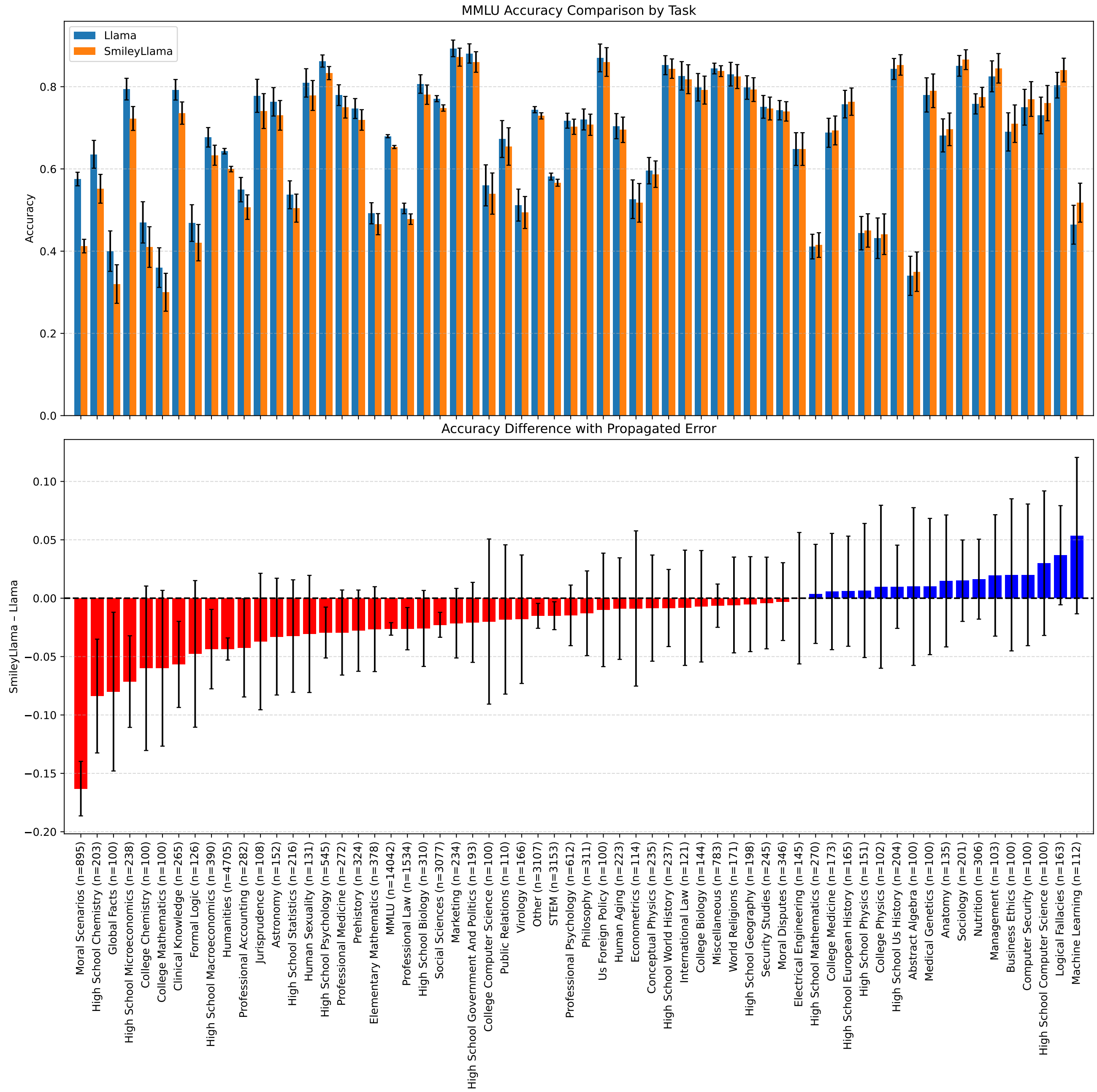

Supplementary Figure 7: *A visualization of how the SmileyLlama SFT procedure affects zero-shot Massive Multitask Language Understanding (MMLU) score[2]. There were no technical replicates performed, since the accuracy was assessed through loglikelihoods for each answer, which are deterministic. All bars are mean, and error bars are standard error (top) and propagated standard error (bottom)* (top) Generally, MMLU scores decrease with SFT. One might expect that fine-tuning which improves the ability to generate molecules may translate to improved performance on chemistry, but this was found to be the opposite of the case; chemistry-specific sections of the MMLU saw among the worst degradation in score after SFT. Anecdotally, SmileyLlama will often respond to a chemistry-related query with a SMILES string. The "moral scenarios" section of the MMLU had the largest difference in score between the base and instruct Llama models; while SmileyLlama scores about 41% compared to Llama-3.1-8B Instruct's 57%, the base model Llama-3.1-8B score centers around 28%. This may be due to much of the "moral knowledge" coming during post-training. On the other hand, there are some tasks, such as "Machine Learning", where performance appears to improve, although the standard error bars for each of these tasks include the mean so we cannot conclusively say that the SFT procedure used for SmileyLlama improved Llama's abilities outside of SMILES string generation. Error bars are given as standard error in the upper figure and propagated standard error in the second, respectively.

Supplementary Figure 8: *Example of code generation.*

```
Input:  Write me some python code which calculates the n'th Fibonacci number
Output:
```

```
def fibonacci(n):
    if n <= 0:
        return "Input should be a positive integer"
    elif n == 1:
        return 0
    elif n == 2:
        return 1
    else:
        a, b = 0, 1
        for _ in range(2, n):
            a, b = b, a + b
        return b
```

References

[1] Dubey, A. et al. The Llama 3 Herd of Models. `http://arxiv.org/abs/2407.21783`.

[2] Hendrycks, D.; Burns, C.; Basart, S.; Zou, A.; Mazeika, M.; Song, D.; Steinhardt, J. Measuring Massive Multitask Language Understanding.
